# Supplementary material for: Food anaphylaxis diagnostic marker compilation in machine learning design and validation
Source: PLoS One. 2023 Apr 5;18(4):e0283141. doi: 10.1371/journal.pone.0283141 (PMC10075449; doi:10.1371/journal.pone.0283141)
Supplement: Attachment — Submitted filename: PONE-D-22-23228_reviewer.pdf [file pone.0283141.s001.pdf]

PLOS ONE

Food Anaphylaxis Diagnostic Marker Compilation in Machine Learning Design and Validation

--Manuscript Draft--

**Manuscript Number:**	PONE-D-22-23228
**Article Type:**	Research Article
**Full Title:**	Food Anaphylaxis Diagnostic Marker Compilation in Machine Learning Design and Validation
**Short Title:**	Food Anaphylaxis Diagnostic Marker Compilation in Machine Learning Design and Validation
**Corresponding Author:**	Inderpal S Randhawa, MD<br>Translational Pulmonary and Immunology Research Center<br>Long Beach, CA UNITED STATES
**Keywords:**	machine learning<br>food anaphylaxis<br>artificial intelligence
**Abstract:**	Background<br><br>Traditional food allergy assessment of anaphylaxis remains limited in accuracy and accessibility. Current methods of anaphylaxis risk assessment are costly with low predictive accuracy. The Tolerance Induction Program (TIP) for anaphylactic patients undergoing TIP immunotherapy produced large-scale diagnostic data across biosimilar proteins, which was used to develop a machine learning model for patient-specific and allergen-specific anaphylaxis assessment. In explanation of construct, this work describes the algorithm design for assignment of peanut allergen score as a quantitative measure of anaphylaxis risk. Secondarily, it confirms the accuracy of the machine learning model for a specific cohort of food anaphylactic children.<br><br>Methods and results<br><br>Machine learning model design for allergen score prediction utilized 247 individual allergy assays per patient. Accumulation of data across total IgE subdivision served as the basis of data organization. Two regression based Generalized Linear Models (GLM) were utilized to position allergy assessment on a linear scale. The initial model was further tested with sequential patient data over time. A Bayesian method was then used to improve outcomes by calculating the adaptive weights for the results of the two GLMs of peanut allergy score prediction. A linear combination of both provided the final hybrid machine learning prediction algorithm. Specific analysis of peanut anaphylaxis within one endotype model is estimated to predict the severity of possible anaphylactic reaction to peanut with a recall of 98.3% on a dataset of 498 juvenile patients with various food allergies, including but not limited to peanut allergy. Receiver Operating Characteristic analysis yielded over 94% AUC results within peanut allergy prediction.<br><br>Conclusions<br><br>Machine learning algorithm design established from comprehensive molecular allergy data produces high accuracy and recall in anaphylaxis risk assessment. Subsequent design of additional food protein anaphylaxis algorithms is needed to improve the precision and efficiency of clinical food allergy assessment and immunotherapy treatment.
**Order of Authors:**	Inderpal S Randhawa, MD
	Greg Sigalov
	Kirill Groshenkov
**Additional Information:**	
**Question**	**Response**

**Financial Disclosure**<br><br>Enter a financial disclosure statement that describes the sources of funding for the work included in this submission. Review the submission guidelines for detailed requirements. View published research articles from *PLOS ONE* for specific examples.<br><br>This statement is required for submission and **will appear in the published article** if the submission is accepted. Please make sure it is accurate.<br><br>**Unfunded studies**<br>Enter: *The author(s) received no specific funding for this work.*<br><br>**Funded studies**<br>Enter a statement with the following details:<br>• Initials of the authors who received each award<br>• Grant numbers awarded to each author<br>• The full name of each funder<br>• URL of each funder website<br>• Did the sponsors or funders play any role in the study design, data collection and analysis, decision to publish, or preparation of the manuscript?<br>• **NO** - Include this sentence at the end of your statement: *The funders had no role in study design, data collection and analysis, decision to publish, or preparation of the manuscript.*<br>• **YES** - Specify the role(s) played.<br><br>* typeset	The authors received no specific funding for this work.
**Competing Interests**<br><br>Use the instructions below to enter a competing interest statement for this submission. On behalf of all authors, disclose any competing interests that could be perceived to bias this work—acknowledging all financial support and any other relevant financial or non-financial competing interests.<br><br>This statement is required for submission	The authors have declared no competing interests exist.

and **will appear in the published article** if the submission is accepted. Please make sure it is accurate and that any funding sources listed in your Funding Information later in the submission form are also declared in your Financial Disclosure statement.<br><br>View published research articles from *PLOS ONE* for specific examples.<br><br>**NO authors have competing interests**<br><br>Enter: *The authors have declared that no competing interests exist.*<br><br>**Authors with competing interests**<br><br>Enter competing interest details beginning with this statement:<br><br>*I have read the journal's policy and the authors of this manuscript have the following competing interests: [insert competing interests here]*<br><br>* typeset	
**Ethics Statement**<br><br>Enter an ethics statement for this submission. This statement is required if the study involved:<br><br>• Human participants<br>• Human specimens or tissue<br>• Vertebrate animals or cephalopods<br>• Vertebrate embryos or tissues<br>• Field research<br><br>Write "N/A" if the submission does not require an ethics statement.<br><br>General guidance is provided below. Consult the submission guidelines for detailed instructions. **Make sure that all information entered here is included in the Methods section of the manuscript.**	All patient data utilized in this study was obtained by informed consent through Advarra Institutional Review Board.

Powered by Editorial Manager® and ProduXion Manager® from Aries Systems Corporation

**Format for specific study types**<br><br>**Human Subject Research (involving human participants and/or tissue)**<br>• Give the name of the institutional review board or ethics committee that approved the study<br>• Include the approval number and/or a statement indicating approval of this research<br>• Indicate the form of consent obtained (written/oral) or the reason that consent was not obtained (e.g. the data were analyzed anonymously)<br><br>**Animal Research (involving vertebrate animals, embryos or tissues)**<br>• Provide the name of the Institutional Animal Care and Use Committee (IACUC) or other relevant ethics board that reviewed the study protocol, and indicate whether they approved this research or granted a formal waiver of ethical approval<br>• Include an approval number if one was obtained<br>• If the study involved *non-human primates*, add *additional details* about animal welfare and steps taken to ameliorate suffering<br>• If anesthesia, euthanasia, or any kind of animal sacrifice is part of the study, include briefly which substances and/or methods were applied<br><br>**Field Research**<br><br>Include the following details if this study involves the collection of plant, animal, or other materials from a natural setting:<br>• Field permit number<br>• Name of the institution or relevant body that granted permission	
**Data Availability**<br><br>Authors are required to make all data underlying the findings described fully available, without restriction, and from the time of publication. PLOS allows rare exceptions to address legal and ethical concerns. See the PLOS Data Policy and FAQ for detailed information.	Yes - all data are fully available without restriction

A Data Availability Statement describing where the data can be found is required at submission. Your answers to this question constitute the Data Availability Statement and **will be published in the article**, if accepted.<br><br>**Important:** Stating 'data available on request from the author' is not sufficient. If your data are only available upon request, select 'No' for the first question and explain your exceptional situation in the text box.<br><br>Do the authors confirm that all data underlying the findings described in their manuscript are fully available without restriction?	
**Describe where the data may be found in full sentences. If you are copying our sample text, replace any instances of XXX with the appropriate details.**<br><br>• If the data are **held or will be held in a public repository**, include URLs, accession numbers or DOIs. If this information will only be available after acceptance, indicate this by ticking the box below. For example: *All XXX files are available from the XXX database (accession number(s) XXX, XXX.).*<br>• If the data are all contained **within the manuscript and/or Supporting Information files**, enter the following: *All relevant data are within the manuscript and its Supporting Information files.*<br>• If neither of these applies but you are able to provide **details of access elsewhere**, with or without limitations, please do so. For example:<br><br>*Data cannot be shared publicly because of [XXX]. Data are available from the XXX Institutional Data Access / Ethics Committee (contact via XXX) for researchers who meet the criteria for access to confidential data.*<br><br>*The data underlying the results presented in the study are available from (include the name of the third party*	Data utilized in machine learning developed is available on a secure cloud server.

and contact information or URL).<br>• This text is appropriate if the data are owned by a third party and authors do not have permission to share the data.<br><br>* typeset	
Additional data availability information:	

Manuscript
Click here to access/download;Manuscript;AI manuscript clean.docx

Food Anaphylaxis Diagnostic Marker Compilation in Machine Learning Design and Validation

Inderpal Randhawa, MD, Kirill Groshenkov, Greg Sigalov, PhD

INTRODUCTION

Food allergy and anaphylaxis remain a high prevalence disorder in developed countries . With an estimated 6% of children diagnosed with food anaphylaxis, the diagnosis burden encompasses both patients and families . The cost of living with food anaphylaxis is $24.8 (95% CI, $20.6-$29.4) billion annually ($4184 per year per child) . Of greater concern, the incidence of food allergy is increasing in both developed and developing countries

Proper diagnosis of food anaphylaxis has evaded singular test methods. The classical method of skin prick testing at best maintains 50-60% positive predictive value . Serum Immunoglobulin E (sIgE) testing via older RAST methods or more recent Immunocap technology maintains over 50% false positivity in food allergy diagnosis . In the last two decades, component resolved diagnostic testing held promise in greater anaphylaxis predictive accuracy. However, large studies reveal limitations in any improvement of accuracy of sIgE . The gold standard of diagnosis remains the double-blind placebo controlled food challenge (DBPCFC). Interestingly, even patients who pass a DBPCFC are able re-develop anaphylaxis to that “passed” food challenge protein .

Our institute has developed a novel approach to the predictive diagnosis of food anaphylaxis followed by induction of sustained unresponsiveness of primary food allergens. The Tolerance Induction Program (TIP) involves utilization of large-scale data analytics assessing hundreds of pro allergic markers (skin prick testing, component resolved diagnostics, immunocap specific IgE, histamine release assay, peripheral eosinophil count) as well as tolerance markers (cytokine profiles, IgG4 specifics, total IgG4, total IgG) across collected patient cohorts since 2007. The machine learning process organizes an incoming patient’s specific markers against the molecular groupings of the database. Once analyzed, the patient’s markers are assessed into an endotype and a subsequent risk assessment of food allergens and biosimilar proteins is organized. This organized “snapshot” of an individual patient is further utilized to build food protein dose exposure cycles based on standardized eliciting doses of food proteins. Repeat marker analysis is monitored to assess the final process of tolerance induction and one-week sustained unresponsiveness for all primary allergens. The TIP model has been previously published.9

The goal of this study is to overcome the limitations of current food allergy predictive models. The endpoint of TIP is high dose food allergen protein exposure on a minimum of a weekly basis with continued reduction in biosimilar food allergy markers. The severity of allergic reaction is quantitatively characterized by an allergen score. The databases and extracted formats to achieve these results served as the basis for machine learning development. A machine learning algorithm was developed to predict the allergen score using lab test data as features. The described algorithm was built and optimized to achieve > 95% recall. The training and testing data model are further described specific to clinical outcomes defined as sustained unresponsiveness.

MATERIALS AND METHODS

Data

Data sets are organized into two primary groups. The first group involves all molecular data which was consistently acquired from each patient enrolled in the program since 2007. The data set includes extended profiles of component resolved diagnostics, skin prick testing, and sIgE to all primary plant and animal protein regardless of the patient's clinical history. The second data set includes immune system markers consistently acquired from each patient enrolled in the program since 2007. Markers include B cell antibody diversity, cytokine production, basophil indices, eosinophil counts, and lymphocyte counts. The final data set includes the similarity of plant and animal species allergens utilizing data adopted from public domains such as the food allergen datasets Allergen Nomenclature (http://www.allergen.org/index.php, accessed on 9 October 2020) and the Structural Database of Allergenic Proteins (SDAP) (http://fermi.utmb.edu/ SDAP/). All datasets have been rigorously screened, and there is no duplication between positive and negative samples.

A Machine Learning Algorithm for Allergen Score Prediction

To provide a physician with a tool for quick and accurate evaluation of the patient's risk of anaphylaxis and the severity of a possible allergic reaction to peanut, we developed a Machine Learning (ML) algorithm. The algorithm is using a set of lab test data with up to 247 individual tests, as well as the patient's personal information such as age and weight, as feature candidates. In the process of model optimization and training, relevant features are selected via iterations of a greedy selection algorithm, while features not meeting the standard relevance criteria are dropped. This automatic, data-centric process eliminates a human factor in deciding which test results should or should not be used in the ML model design. The larger set of tests for possible allergic proteins is available, the higher accuracy of the ML algorithm could theoretically be achieved. The relevant features are assigned adoptive weights as the result of model training. The training data set is tabular, based on depersonalized patient data, and will be described in detail below. Once trained, the ML model is used for new (out of set) patients to predict a quantified expected patient's allergic reaction to peanut. The physician may then consider the predicted value while finalizing the diagnosis and selecting the treatment.

The severity of allergic reaction to peanut in a particular patient is normally classified as Anaphylactic (Ana), Sensitized (Sen), or Tolerant (Tol). Additionally, within each of these three classes, it is characterized by a positive number up to 100 referred to as an allergen score. The larger the number, the stronger the allergic reaction and, consequently, the risk of anaphylaxis. The score is usually rounded so that there could be up to 5 discrete severity levels within each severity class, for example Ana20, Ana40, Ana60, Ana80, Ana100. Since, for example, Sen100 is the highest severity level in the class Sen, and Ana20 is the lowest severity level in the class Ana that is directly above class Sen, it is natural to use a continuous absolute linear scale from 0 to 300 for the allergen score. Scores up to 100 correspond to identical scores within class Tol; scores between 120 to 200 correspond to Sen20 … Sen100; and finally, scores between 220 to 300 correspond to Ana20 … Ana100. These score representations are equivalent, each having its own advantages. In what follows, we will use both notations interchangeably.

To be clear, the ML algorithm is used as an auxiliary recommendation tool to assist a qualified human medical professional, who may consider the algorithm's prediction in a clinical decision making process ultimately left to the physician. Given the severity of anaphylaxis, and the fact that it is safer to overestimate rather than underestimate the risk of anaphylaxis, the algorithm is optimized, through its

objective function’s design, to have the highest accuracy for class Ana and the upper levels of class Sen, and to err toward higher allergen scores rather than uniformly. In technical terms, positive errors (risk overestimation) have smaller weights in weighted Root Mean Square Error (RMSE) summation than negative errors do.

Dataset and Features

For the purposes of this paper, we only focus on peanut allergy for endotype 1 patients. However, the same approach is applicable to other proteins and endotypes (results to be published elsewhere). The selected dataset included 498 patients, out of which 310 had total IgE < 1000 kU/L and 188 had total IgE > 1000 kU/L, thus representing two cohorts with different levels of susceptibility to food allergies. Figure 1 shows the distribution of total IgE values for all patients whose data were used in this study.

The available dataset consists of depersonalized patient profiles along with the results of various laboratory tests performed before and during treatment. Those data provide the (potential) input features (independent variables, also called predictors) for a predictive ML algorithm. For each patient, three types of data are available:

- patient’s personal information, including gender, age, and weight;
- the results of up to 247 blood and skin lab tests performed;
- peanut allergy severity classes and scores as determined by a qualified medical professional (these are the dependent variables whose ground truth values are used to train the ML model).

The lab test results are usually represented by a nonnegative number within a certain range, with some notable exceptions. First of all, certain features cannot be measured reliably near the lower and/or upper limits of their range; such results may be shown as “less than” or “greater than” a certain threshold value, with a numerical value in the intermediate range where an accurate measurement is possible. Secondly, some of the tests have Boolean or integer-valued underlying nature and could be better treated as binary, categorical, or ordinal variables, rather than as continuous variables. Lastly, the values for many tests are missing, usually because those tests were deemed (by a physician) redundant or unnecessary, considering the patient’s anamnesis. Thus, only the ML methods that are capable of dealing efficiently with massively missing data should be considered for the algorithm. Fig. 2 illustrates the range and distribution of values for some representative features.

Algorithm Design

As noted above, there are two ways to characterize the allergen score: either as a class (Tol, Sen, Ana) and further as a level 20, 40, …, 100 within that class (e.g., Ana40), or as a continuous variable from 0 to 300, rounded to the nearest 20. The step of 20 is chosen for convenience only and does not affect the

algorithm as such. Accordingly, there are two possibilities for the design of a predictive ML algorithm. To reflect the former representation, one could build a cascade of a 3-class classifier and a regression model for the level within the class. For the latter representation, a regression model for the continuous score that spans all three classes could be constructed.

For the former choice, to assign one of the 3 classes, one could take into account the fact that those classes are actually ordered (Tol < Sen < Ana in terms of the severity of the allergic reaction), and thus use ordinal regression. Alternatively, one could ignore that fact and use pure classification into categorical variables, expecting that the ML model would learn on its own to assign classes in the correct order. In what follows, we will generate ML models based on each of those design solutions and compare their outcomes.

We began by creating a regression-based Generalized Linear Model (GLM) with link functions to mimic the nonlinear behavior or a hypothesized type of distribution (normal or Poisson distribution, as indicated by the data analysis) for some of the features. We chose a continuous score ranging from 0 to 300 as the dependent variable and rounded the obtained real-numbered predictions to the nearest 20.

Based on biomedical information, certain features are known a priori to be possibly relevant for peanut allergy prediction. The GLM regression model is initially trained using those a priori features, while estimating their actual relevance. After an automatic feature elimination process via iterations of a greedy algorithm, the relevant features are found to be peanut_ige, peanut_igg4, peanut_spt, arah_1, arah_2, arah_3, arah_8, arah_9. While higher numerical values of these features usually imply a higher peanut allergy score, the dependence of the latter on the features is non-trivial and extremely noisy, as illustrated by Fig. 3, and requires an advanced ML model to allow for meaningful prediction.

The model generated using the abridged (known a priori) set of features might be improved by considering additional features provided by lab tests for proteins not obviously biosimilar to peanut, and thus not deemed a priori relevant. The model is therefore trained further, trying one additional candidate feature at a time (out of about 240 available features, including patient's age and other personal information). A feature that improves the model is preserved, and then another iteration over candidate features is performed, until no further improvement can be achieved. Some examples of features that are not directly related to peanut but still improve the ML model that predicts the peanut allergy score are total_igg4, cyt, and eos (Eosinophil count). Table 1 shows how addition of these non-obvious features, selected automatically through greedy incremental feature testing and addition or rejection, improves the model. The prediction error is estimated using the Leave-One-Out (LOO) cross-validation method.

The mean absolute error (MAE) of the above models is well under 20, which is the distance between the adjacent values of the allergy score's ground truth values. This means that the ML algorithm predicts the allergy severity level to within one step of the ladder, on average. However, it might not always predict

the correct class (Ana, Sen, or Tol) when the ground truth level is at or near the boundary between classes (100 or 200). For example, it might predict level Sen100 when the ground truth is Ana20, or vice versa. While the numerical difference between these scores is still only 20, the medical implications of the misclassification (Sen vs Ana) may be serious. We addressed this issue by predicting the class first, and the level within each class second. We compared two approaches for that classification task, one through pure classification and the other through ordinal regression, in which the information about class ordering is used extensively. In both models, we used tree-based methods trained with gradient boosting. The ordinal regression method turned out to be more accurate and was selected for this task. The performance of this method is shown in Table 2. Note that there were no patients tolerant to peanut on the chosen dataset, therefore only classes Ana and Sen are present in the results. However, the ordinal regression method we used is not limited to 2-class classification as it is not a binary or logistic classification by its nature. Classification into 3 (or more, for that matter) classes may be effectively performed using the same method for other food proteins or groups of patients.

Finally, we trained the GLM regression model described above on the data for each class separately, thus achieving both a more accurate classification and more accurate prediction of the level with the class. However, the original full-range (0 to 300) GLM regression model still could be used to improve the final outcomes, because it is trained on a larger dataset. We combined both GLMs by using a Bayesian method to calculate the adaptive weights for the results of the two methods of peanut allergy score prediction. A linear combination of them provided the final hybrid ML prediction algorithm. Its design is summarized in Figure 4.

The accuracy of the final ML algorithm was characterized with ROC curves. To verify that the algorithm performs well for both highly susceptible and less susceptible patients, we calculated the ROC curves for 310 patients with total IgE < 1000 and 188 patients with total IgE > 1000. The results shown in Fig. 5 demonstrate that the proposed ML algorithm could be used as a diagnostic support tool for a physician to evaluate the expected severity of patient's allergic response to peanut.

DISCUSSION:

Machine learning algorithm design in modern medicine largely endorses imaging aspects of medical specialties such as radiology and ophthalmology. ML accuracy and recall in imaging associated medical specialties continue to improve as reflected in the regulatory approval of such devices. The specialty of Allergy and Immunology to date has no diagnostic machine learning algorithm published in any of the atopic disease states: eczema, asthma, rhinitis or food anaphylaxis. The Tolerance Induction Program has structured its clinical therapeutic model upon data collection across immunological, phylogenetic and clinical markers. The totality of these markers are organized to study food anaphylaxis patients into specific endotype cohorts. Once identified, the cohort specific analysis utilizes machine algorithms based on aforementioned data sets to determine food protein specific anaphylaxis risk. Furthermore, the risk assessment produced serves as the basis for additional machine learning systems producing

food protein based immunotherapy schedules on a patient specific basis. Ultimately, the risk assessment is re-calculated per annum as the patient progresses through large challenges of food proteins in challenge sequences. Upon completion, the patient consumes between 10-30 grams of food specific protein in one sitting to all of once anaphylactic foods. In subsequent weeks, the consumption of this large amount of food protein occurs on a weekly or biweekly basis thus achieving sustained unresponsiveness while freely consuming once anaphylactic foods.

Our machine learning algorithm is designed utilizing anaphylaxis clinical, diagnostic and eliciting dose data. Such data in an anaphylaxis cohort is critical as it reflects known risk of the disease in a large cohort. The ML algorithm design was further specified to peanut. Focus on peanut was necessary as it was one of the most common food anaphylactic proteins in the study population. The specific analysis of peanut anaphylaxis within this one endotype model was estimated to predict the severity of possible anaphylactic reaction to peanut with a recall of 98.3% on a dataset of 498 juvenile patients with various food allergies. The validation of the ML algorithm was characterized with ROC curves. Calculation of ROC curves for 310 patients with total IgE < 1000 kU/L and 188 patients with total IgE > 1000 kU/L yielded over 94% AUC results within peanut allergy prediction. The results demonstrate that the proposed ML algorithm could be used as a diagnostic support tool for a physician to evaluate the expected severity of patient's allergic response to peanut. Moreover, it supports the accuracy of peanut anaphylaxis assessment in targeted treatment in the Tolerance Induction Program.

Machine learning algorithm design established from comprehensive molecular allergy data produces high accuracy and recall in anaphylaxis risk assessment. Further design of additional food protein anaphylaxis algorithms is needed to improve the precision and efficiency of clinical food allergy assessment and immunotherapy treatment.

REFERENCES

1. Datema M, Lyons SA, Fernández-Rivas M, Ballmer-Weber, B, et al. Estimating the Risk of Severe Peanut Allergy Using Clinical Background and IgE Sensitization Profiles. *Frontiers in Allergy*. 2021 Jan. 2(1): 670-789.

2. Gupta R S, Warren C M, Smith BM, Blumenstock JA, et al. The Public Health Impact of Parent-Reported Childhood Food Allergies in the United States. *Pediatrics*. 2018 Jul. 142(6).

3. Gupta R, Holdford D, Bilaver L, Dyer A, et al. The economic impact of childhood food allergy in the United States. *JAMA Pediatrics.* 2013 Dec. 167(11), 1026–1031.

4. Tang ML, Mullins RJ. Food allergy: is prevalence increasing?. *Internal Medicine Journal*. 2017 Jun. 47(3), 256–261.

5. Soares-Weiser K, Takwoingi Y, Panesar SS, Muraro A, et al. EAACI Food Allergy and Anaphylaxis Guidelines Group. The diagnosis of food allergy: a systematic review and meta-analysis. *Allergy*. 2014 May. 69(1), 76–86.

6. Wood R A, Segall N, Ahlstedt S, Williams PB. Accuracy of IgE antibody laboratory results. *Annals of Allergy, Asthma & Immunology.* 2007 Nov. 99(1), 34–41.

7. Foong RX, Dantzer JA, Wood RA, Santos AF. Improving Diagnostic Accuracy in Food Allergy. *Journal of Allergy and Clinical Immunology. In practice*. 2021 July. 9(1), 71–80.

8. Fleischer DM, Conover-Walker MK, Christie L, Burks AW, et al. Peanut allergy: recurrence and its management. *Journal of Allergy and Clinical Immunology*. 2004 Feb. 114(5), 1195–1201.

9. Randhawa I. Tolerance Induction Program Effect Explains Variation in Wheal Size, sIgE, and IgG4 in Peanut Allergic Children. *Journal of Allergy & Therapy*. 2019 July. 10 (2):1-10.

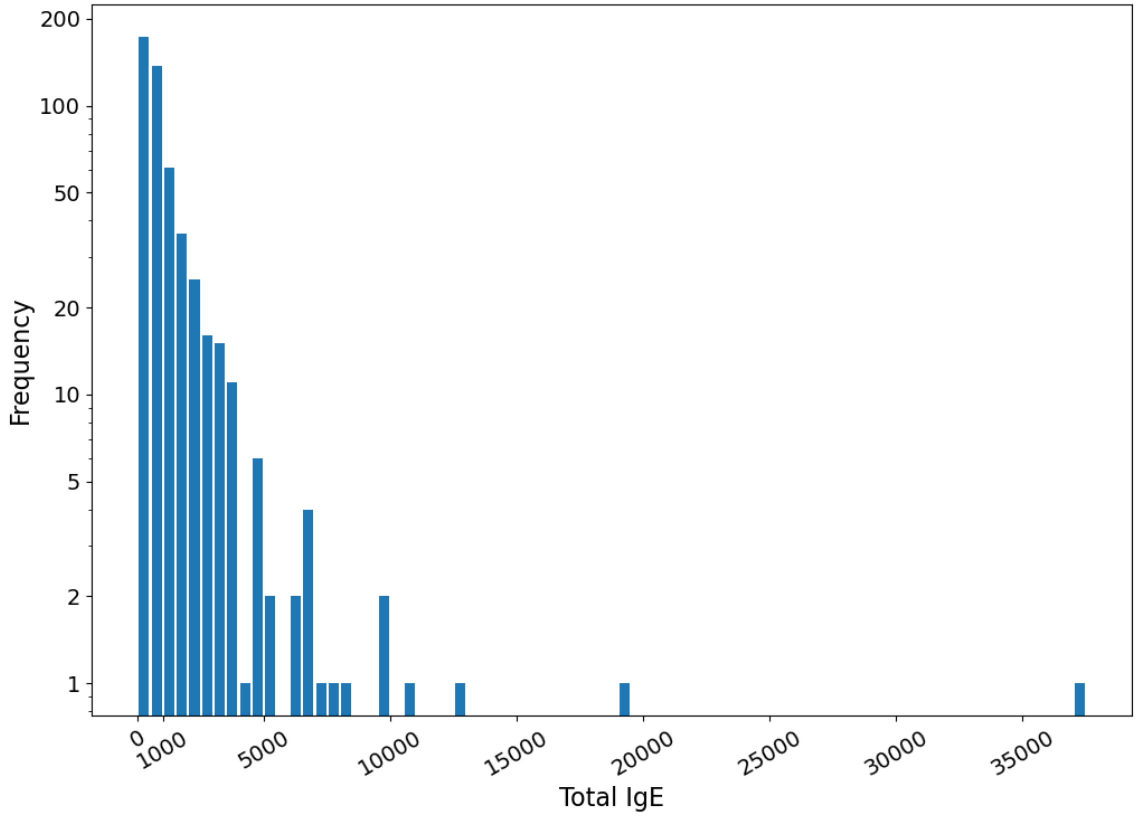

Figure 1. Distribution of total IgE values over the selected patient dataset.

 Click here to access/download;Figure;Figure 2.docx

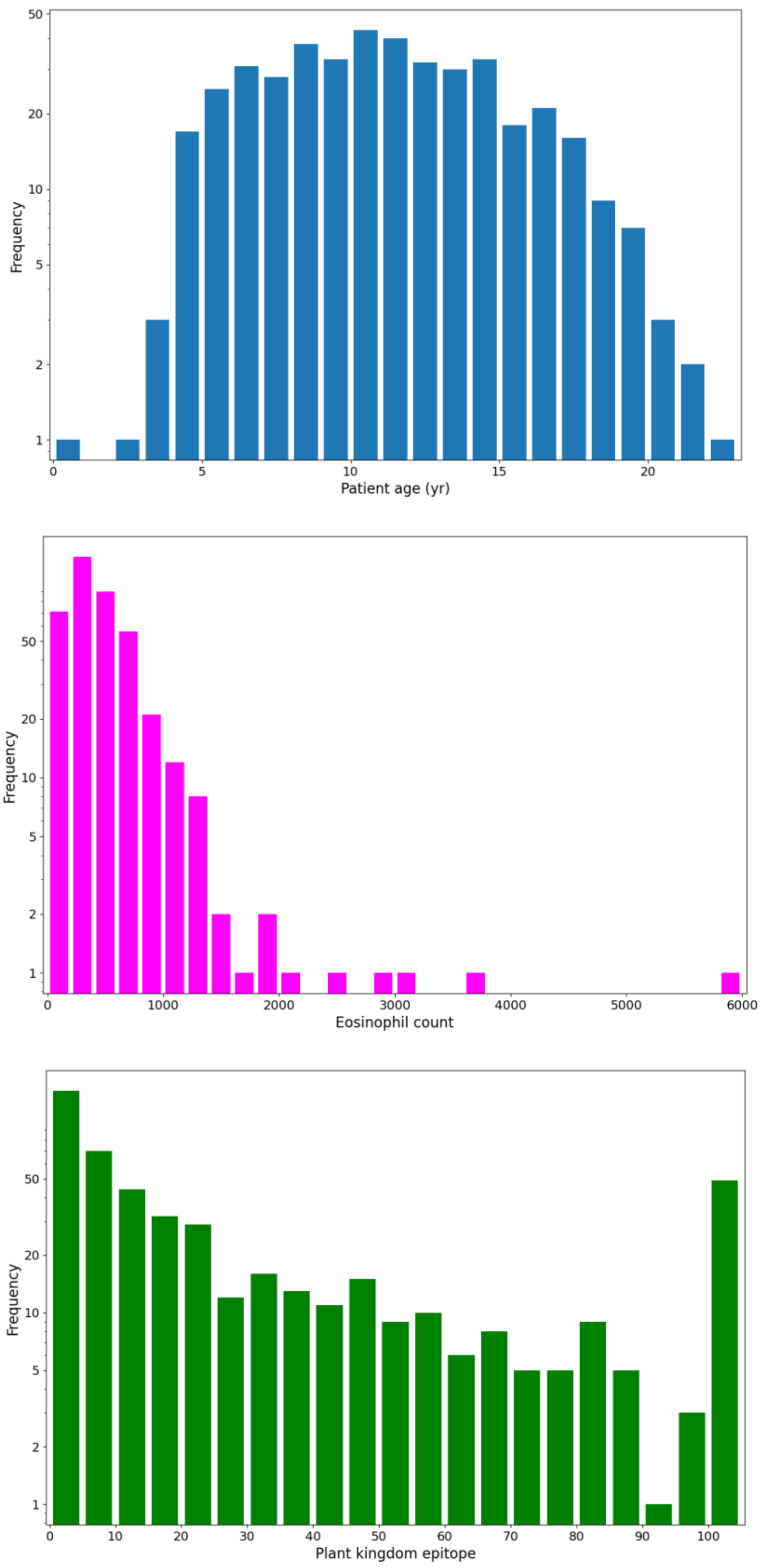

Figure 2. Distribution of values for some data features.

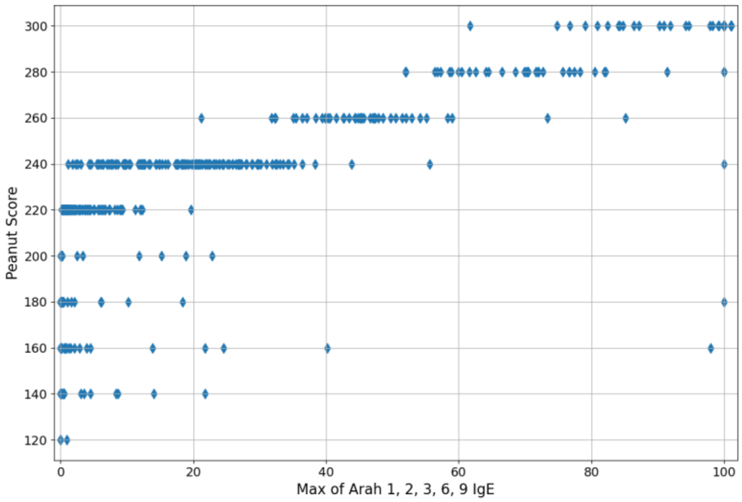

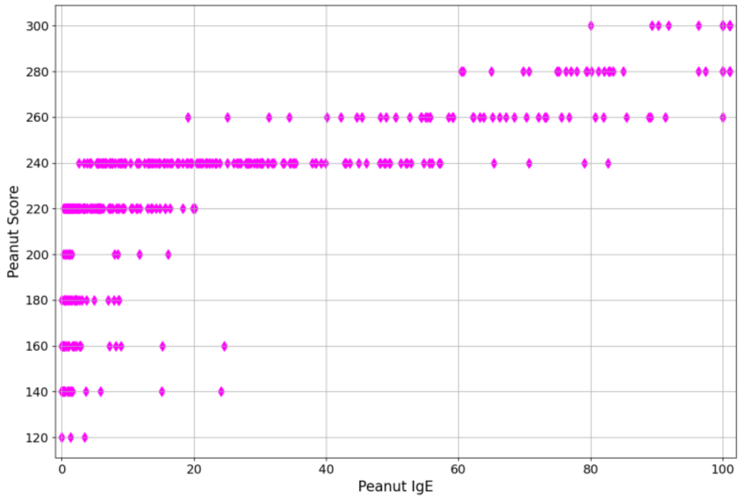

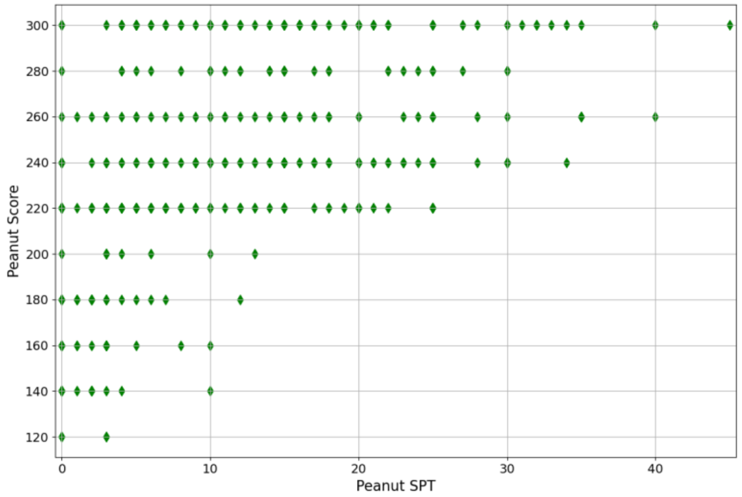

Figure 3. Correlation between the dependent variable (peanut allergy score) and some relevant predictors. Absolute peanut allergy scores 140-200 can be interpreted as Sen40 to Sen100, and scores 220-300 as Ana20 to Ana100. By design, arahs = max(arah_1, …, arah_9).

 Click here to access/download;Figure;Figure 4.docx

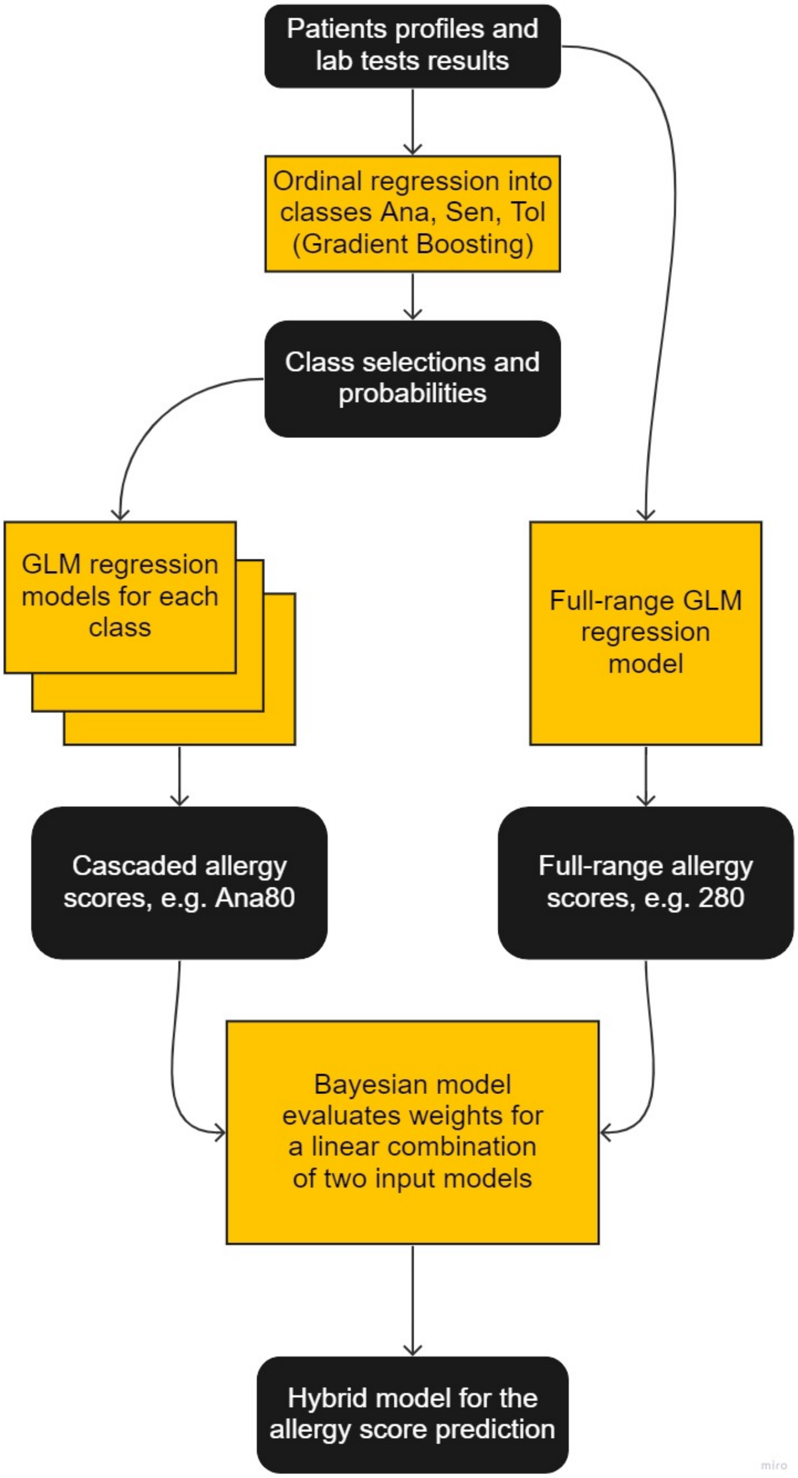

Figure 4. The design of the proposed ML algorithm for prediction of the peanut allergy score.

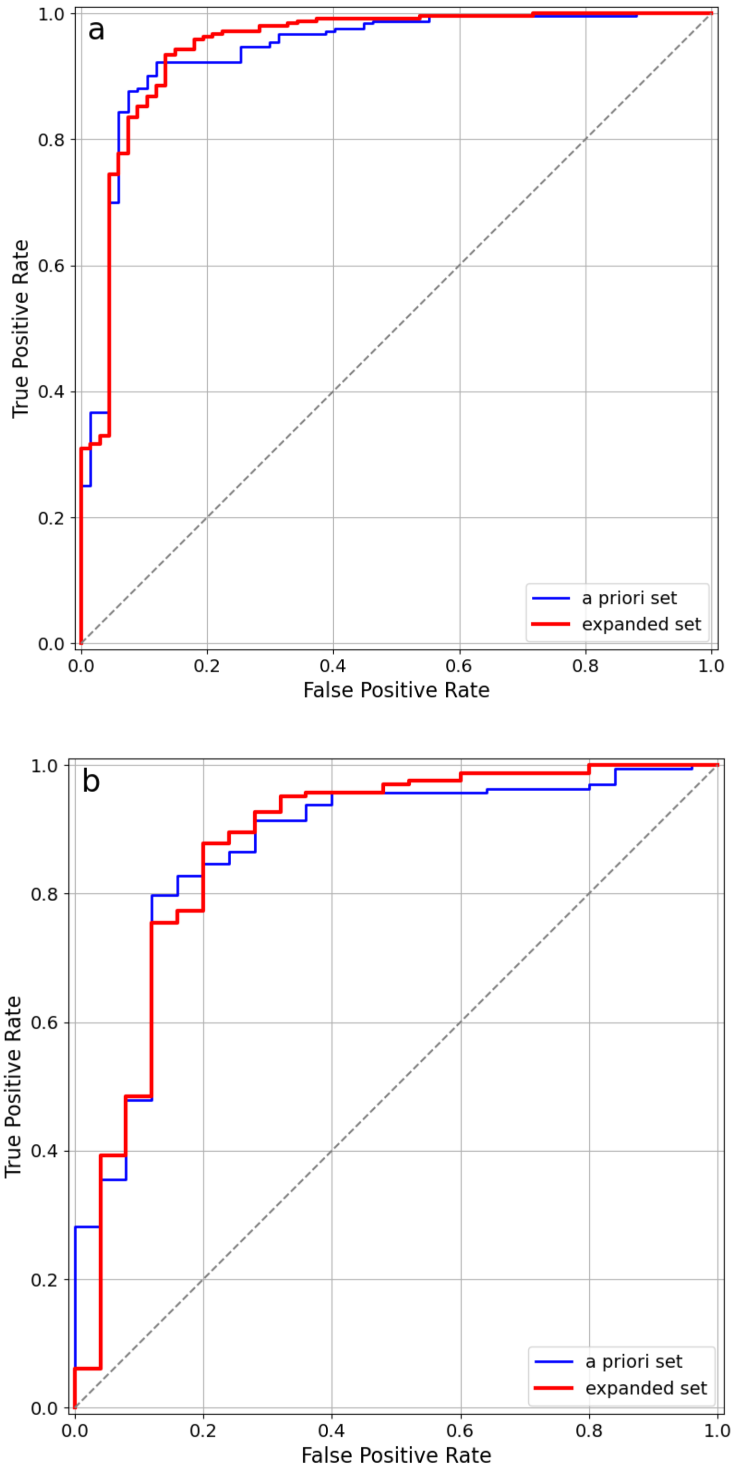

Figure 5. ROC curves for the proposed ML algorithm's prediction that the patient is Anaphylactic (Ana) vs Sensitive (Sen): (a) patients with total IgE < 1000 kU/L, AUC 0.944; (b) patients with total IgE > 1000 kU/L, AUC 0.874 (AUCs are given for the expanded feature sets).
