# Supplementary material for: Food anaphylaxis diagnostic marker compilation in machine learning design and validation
Source: PLoS One. 2023 Apr 5;18(4):e0283141. doi: 10.1371/journal.pone.0283141 (PMC10075449; doi:10.1371/journal.pone.0283141)
Supplement: Attachment — Submitted filename: Response to Reviewers.docx [file pone.0283141.s002.docx]

Response to the Editor and the Reviewers

I. Response to the Editor (re: Journal Requirements)

1. Completed.

2. (a)	**Q**: In the Methods section of your revised manuscript, please include the full name of the institutional review board or ethics committee that approved the protocol, the approval or permit number that was issued, and the date that approval was granted.

**A**: The study was approved under Advarra Institutional Review Board (IRB #PRO00043361). The approval date was September 19, 2020.

2. (b)	The complete codes and data necessary to reproduce our calculations and verify our findings are available at <https://github.com/TPIRC/ai_paper_2022> (a public repository).

3.	**Q**: Please provide additional details regarding participant consent. In the ethics statement in the Methods and online submission information, please ensure that you have specified what type you obtained (for instance, written or verbal, and if verbal, how it was documented and witnessed). If your study included minors, state whether you obtained consent from parents or guardians. If the need for consent was waived by the ethics committee, please include this information.

**A**: Under the IRB approval, all parents and/or patients consented to participate in the Tolerance Induction Program including the written consent to submit patient data as part of research activities.

4. The complete codes and data necessary to reproduce our calculations and verify our findings are available at <https://github.com/TPIRC/ai_paper_2022> (a public repository).

5. Completed.

6. Completed.

7.	**Q**: Please include your full ethics statement in the ‘Methods’ section of your manuscript file. In your statement, please include the full name of the IRB or ethics committee who approved or waived your study, as well as whether or not you obtained informed written or verbal consent. If consent was waived for your study, please include this information in your statement as well.

**A**. The study was approved under Advarra Institutional Review Board (IRB #PRO00043361).

8. Completed.

II. Response to Reviewer #1

1) As described in the section “Algorithm Design”, we are already using ordinal regression. Pure classification was considered during the research phase, and it was rejected as a less accurate method. Detailed comparison of these two methods in the paper is not warranted as there exist many other methods of Machine Learning besides ordinal regression, which theoretically could be considered. It was not our goal to try all of them and make a rigorous comparison of them.
2) Thank you for this question, we made a list of the most important features and included it in the revised manuscript.
3) We developed a custom code in Python that implemented our proposed algorithm. Yes, it will be made freely accessible through a public github repository available at <https://github.com/TPIRC/ai_paper_2022>.
4) Yes, our dataset includes patients with peanut allergy ranging from severe anaphylactic to mildly sensitive reaction. We should note that the dataset was obtained in the course of treatment of patients with various forms of food allergy (not limited to peanut allergy) in an actual medical clinic, as opposed to a purely academic study of volunteers. For this reason, we have not tested any perfectly healthy individuals within this study. However, we still have a wide range of degrees of peanut allergy among the subjects of the study.
5) This algorithm is currently being deployed for practical use in the Southern California Food Allergy Institute. It is our hope that other medical organizations also use it in the future once this manuscript is published and the method comes to the attention of a wider audience of medical professionals.
6) We made every effort to do so.
7) We are not aware of a public database that would contain the same or wider set of data, compared to the dataset used in our research. If you’d be kind enough to point out such a database that you know of, we would gladly test our algorithm on that independent dataset and include the results in a revised manuscript or a separate publication. Lacking that opportunity, we ensured the robustness of the algorithm using standard methods of Machine Learning such as multi-fold cross-validation.

III. Response to Reviewer #2

1) i. The proposed algorithm is not limited to a linear regression; therefore, the outcome is not simple enough to be presented via a concise mathematical formula. We are using advanced tools of Machine Learning (ML), through which a model is trained on the input data. The trained model (a binary computer file) and a computer code are then used to make a prediction. The mathematical operations that are completed to generate the predictions are too complex to be included in the manuscript, but they are well known to the ML professionals. As such, the explanation of those ML methods is beyond the scope of our manuscript. On the other hand, the list of the most important features and the measure of their importance are included in the revised version of the manuscript. Thank you for asking about them.
   ii. On the other subject, the results of the algorithm’s validation are provided in Table 2 and Figure 5. We ensured the robustness of the algorithm using standard methods of Machine Learning such as multi-fold cross-validation. Once a method has been validated using standard statistical method, the doctor is not supposed to double-check every computer-generated support decision by re-doing the same calculations by hand.
2) i. The anonymized dataset that we used will be made freely available, as required by the Journal. We are sorry if you received an incomplete copy of the manuscript. The tables and figures were submitted along with the manuscript albeit in separate files, as required by the Journal. They are also submitted again with the revised manuscript.
   ii. As we mentioned above, the prediction made by the ML-based model is more complex than the simple algebraic form that you suggested. The calculations require a computer program and cannot be repeated by hand for practical purposes. That program, indeed, calculates the allergy score, which indicates the degree of risk of anaphylaxis on a numerical scale, just as you suggested.
3) The anonymized patient data, obtained and used in this study, and the computer codes necessary to reproduce our calculations and independently verify our findings, are available at <https://github.com/TPIRC/ai_paper_2022> (a public repository).
4) We made every effort to make the language of the manuscript accessible to a wide audience of experts in the field of data science applications in allergy research. The audience for which this paper is intended should have no difficulty with the terminology or standard abbreviations we used. Unfortunately, given the limitations of the journal scope and space, it would not be possible to make this paper an easy reading for every medical practitioner or every computer science professional. Regarding the list of references, we corrected them in the revised manuscript.
5) Thank you for your positive recommendations!
