# Supplementary material for: Food anaphylaxis diagnostic marker compilation in machine learning design and validation
Source: PLoS One. 2023 Apr 5;18(4):e0283141. doi: 10.1371/journal.pone.0283141 (PMC10075449; doi:10.1371/journal.pone.0283141)
Supplement: Attachment — Submitted filename: Rebuttal letter 2.docx [file pone.0283141.s003.docx]

Rebuttal letter

In response to Reviewer #1: on your suggestion, a paragraph was added to the Discussion section in regards to the usage of ordinal vs categorical variables.

In response to Reviewer #2: thank you for pointing out the missing references; they are rectified now. Also, we spelled out the acronyms AUC, RAST, and GLM where appropriate.
