# Supplementary material for: Food anaphylaxis diagnostic marker compilation in machine learning design and validation
Source: PLoS One. 2023 Apr 5;18(4):e0283141. doi: 10.1371/journal.pone.0283141 (PMC10075449; doi:10.1371/journal.pone.0283141)
Supplement: Attachment — Submitted filename: Observation to revision 2.docx [file pone.0283141.s004.docx]

**Dear Editor**

Referring to:

 PONE-D-22-23228R2

**In the next table, you have all my observations regarding the article. Essentially it can be published, but please communicate to the authors the suggestions from observations 1, 2 and 3.**

| No. | Criteria |  |
| --- | --- | --- |
| 1 | The study presents the results of original research. | satisfied,  see obs. 3 |
| 2 | Results reported have not been published elsewhere. | satisfied |
| 3 | Experiments, statistics, and other analyses are performed to a high technical standard and are described in sufficient detail. | satisfied |
| 4 | Conclusions are presented in an appropriate fashion and are supported by the data. | satisfied |
| 5 | The article is presented in an intelligible fashion and is written in standard English. | satisfied |
| 6 | The research meets all applicable standards for the ethics of experimentation and research integrity. | satisfied |
| 7 | The article adheres to appropriate reporting guidelines and community standards for data availability. | satisfied |

**Suggestions** and  **observation to revision 2 for the paper:**

Obs1) We ask the authors to specify if in fig. 1, 2 and 3, on the vertical axis, read the frequency or frequency logarithm. If the frequency logarithm is, please specify the base of the logarithm.

Obs2) It is recommended to control the English language for the entire manuscript because there are still control programs that claim errors and simplest expressions:

1) https://quillbot.com/grammar-check

2) https://www.grammarly.com/gramar-check

Obs3) Plagiaristic check with

https://smallseotools.com/plagiarism-checker/

show that:

1- **Introduction** 13% - plagiarism. Some sources are specified in references, others are not in the reference list. The sources that are not in references can be included, which would also increase the list of references.

2- **Material and Methods, Data** 10% plagiarism. A source not included in the references. Can be included.

3- **A Machine Learning algorithm for allergen score prediction** , 3% plagiarism. . A source not included in the references. Can be included.

Petru Cardei
